# Gluteal Compartment Syndrome following an Iliac Bone Marrow Aspiration

**DOI:** 10.1155/2013/812172

**Published:** 2013-12-11

**Authors:** Edmundo Berumen-Nafarrate, Carlos Vega-Najera, Carlos Leal-Contreras, Irene Leal-Berumen

**Affiliations:** ^1^Orthopaedic Surgery Department, Christus Muguerza del Parque, Calle de la Llave 1419, Office 9, Col. Centro, 31000 Chihuahua, Mexico; ^2^Orthopaedic Surgery Department, Universidad del Bosque, Calle 1345 No. 7b-83, Office 1016, Bogota DC, Colombia; ^3^Faculty of Medicine, Universidad Autónoma de Chihuahua, Circuito Universitario Campus II, Chihuahua, Mexico

## Abstract

The compartment syndrome is a condition characterized by a raised hydraulic pressure within a closed and non expandable anatomical space. It leads to a vascular insufficiency that becomes critical once the vascular flow cannot return the fluids back to the venous system. This causes a potential irreversible damage of the contents of the compartment, especially within the muscle tissues. Gluteal compartment syndrome (GCS) secondary to hematomas is seldom reported. Here we present a case of a 51-year-old patient with history of a non-Hodgkin lymphoma who underwent a bone marrow aspiration from the posterior iliac crest that had excessive bleeding at the puncture zone. The patient complained of increasing pain, tenderness, and buttock swelling. Intraoperative pressure validation of the gluteal compartment was performed, and a GCS was diagnosed. The patient was treated with a gluteal region fasciotomy. The patient recovered from pain and swelling and was discharged shortly after from the hospital. We believe clotting and hematologic disorders are a primary risk factor in patients who require bone marrow aspirations or biopsies. It is important to improve awareness of GCS in order to achieve early diagnosis, avoid complications, and have a better prognosis.

## 1. Introduction

Gluteal compartment syndrome (GCS) is a rare condition that has been scantily reported in the literature. Some case reports have shown that GCS may become a serious complication, as it causes permanent damage to the sciatic nerve, and the myoglobinuria is enough to cause renal failure. This condition is caused by a raise in pressure within the gluteal compartment, caused by prolonged immobilization or other long lasting rest conditions, trauma, drug overdose, or surgical complications in long pelvic trauma or tumor resection procedures [1]. A GCS secondary to hematoma is even less reported than other etiologies commonly related to coagulation disorders such myeloproliferative diseases [2]. Prevention is the key factor, as well as an early diagnosis by a well trained clinician.

## 2. Case Report 

A 51 year-old patient with history of non-Hodgkin lymphoma underwent a bone marrow aspiration at a hospital in another city. Immediately after the procedure, the patient started to bleed in the puncture zone. The patient was treated with compressive sutures at the puncture site and compressive dressings at our hospital. Seven days later he came back to the hospital with acute unbearable pain in his left buttock.

The physical examination showed tenderness and swelling of the left buttock, as well as tenderness on the anterior thigh. The peripheral pulses remained strong, and the sensitivity was normal. We started treatment with lysine clonixinate and fresh frozen plasma, as the patient is allergic to buprenorphine. We performed a gluteal compartment pressure measure that showed a 54 mmHg above the normal threshold. The patient was taken to the operating room immediately, and a gluteal fasciotomy was performed through a posterolateral approach. We found a large gluteal hematoma and blood clots that were cleansed and removed. We started a protocol of IV cephalothin and lysine clonixinate. The patient recovered rapidly after the surgical procedure. However, 24 hours later he had a new episode of acute pain and swelling that required another surgical procedure. We found a small gluteal perforating artery bleeding out of control. Once it was controlled and the bleeding stopped, we close the surgical wound once again.

**Figure 1:**
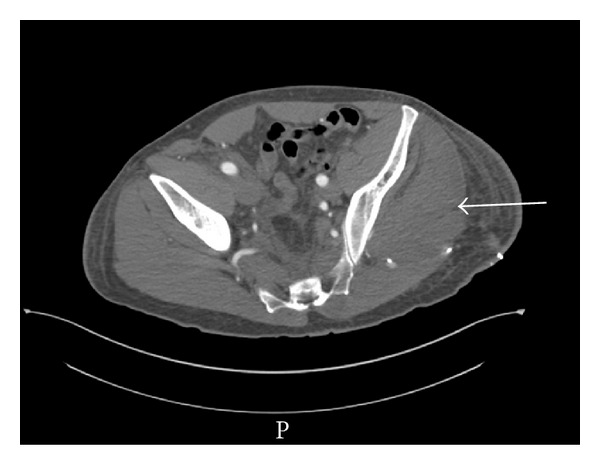
CT scan with IV contrast of the pelvis showing the swollen left gluteal region and the extravasation of contrast media.

A contrast CT scan has been done after 48 hours of being admitted, and it showed diffuse swelling of the left gluteal muscles and the overlying subcutaneous tissue, as well as extravasation of contrast media (Figure 1). The measures of creatine-phosphokinase (CPK) had reached 1001 UI/L.

For the next 6 days the pain and swelling came down progressively, the bleeding from the drain tube stopped, the CPK turned down, and patient had no pain and was discharged.

## 3. Discussion

A compartment syndrome is the result of an increased volume and pressure in certain non expandable musculoskeletal compartments. It is common in the limbs, and its incidence in gluteal compartment is unusual [3]. GCS affects three compartments: the gluteus maximus compartment, where superficial and deep boundaries are represented by the fibrous fascia that is contiguous with the fascia lata of the thigh, the gluteus medius and minimus compartment limited by the wing of the ilium and the combined layers of the fascia lata, with the tensor fascia lata compartment enclosed by its superficial and deep layers [4, 5].

Prolonged local pressure on the gluteal muscles from lying on hard surfaces, usually from surgical positioning in long procedures or in alcohol/drugs abuse neuropsychological blockade situations, the most common causes of GCS [3, 4, 6–8]. There are also some reports of GCS associated with the use of Statin [9, 10]. Trauma and hematomas are rarely reported.

In this report we present a patient with a 4-year diagnosis of a non-Hodgkin lymphoma that developed a GCS as a complication of a posterior iliac crest bone marrow aspiration. This procedure is common procedure in diagnostic and treatment procedures for oncologic diseases [2]. Several complications have been described after an iliac crest bone marrow aspiration, such as infection, needle related incidents, and hemorrhage. Bleeding is probably the most common complication, usually to the gluteal compartment and rarely to the retroperitoneal space [2, 11, 12]. Some of the risk factors that must be considered are coagulation and myeloproliferative disorders, specially in patients under anticoagulant or prophylactic antithrombotic medication such as warfarin [2, 11]. In our case report, our patient had a non-Hodgkin lymphoma, where the platelets level ratio and the clotting times were normal. According to Bain et al. [2], platelet dysfunction seems to be a more important and frequent common risk factor for GCS than thrombocytopenia.

Our diagnosis was clearly determined by the clinical findings and the raise of the compartment pressure of 54 mmHg. Normal values have been reported in 30–45 mmHg [13]. The early diagnosis and the immediate surgical procedure were critical for our good final outcome. Even though GCS is associated with as much as 50% of sciatic nerve impairment [3], our patient did not develop neurological complications. Fatal outcomes have been documented due to acute renal failure following rhabdomyolysis caused by gluteal muscle myonecrosis [4]. In this case we did have an elevated CPK value, but renal function remained normal. We report our experience in this rare case, hoping to create enough awareness for a possible complication in a very common procedure.

## 4. Conclusion

- Patients with clotting disorders, prophylactic antithrombotic medication, or myeloproliferative disorders should be considered at high risk for GCS.
- Iliac crest bone marrow aspiration or biopsy procedures may cause hemorrhagic or compartmental complications.
- Awareness is the key factor, not only in the medical but also in the nursing staff.
- An early diagnosis may prevent severe complications such as an irreversible loss of gluteal muscles, sciatic nerve impairment, or even fatal renal failure due to hemoglobinuria.
- A fasciotomy is a relatively easy procedure that can solve this complication if it is performed on time. In later stages, the outcome might not be that favorable.
